# TβRIII Expression in Human Breast Cancer Stroma and the Role of Soluble TβRIII in Breast Cancer Associated Fibroblasts

**DOI:** 10.3390/cancers8110100

**Published:** 2016-11-04

**Authors:** Bojana Jovanović, Michael W. Pickup, Anna Chytil, Agnieszka E. Gorska, Kimberly C. Johnson, Harold L. Moses, Philip Owens

**Affiliations:** 1 Department of Medical Oncology, Dana-Farber Cancer Institute, Harvard Medical School, Boston, MA 02215, USA; bojana_jovanovic@dfci.harvard.edu; 2 Department of Bioengineering, University of California San Francisco, San Francisco, CA 94117, USA; michael.pickup@ucsfmedctr.org; 3 Department of Cancer Biology, Vanderbilt University, Nashville, TN 37232, USA; anna.chytil@vanderbilt.edu (A.C.); agnes.gorska@vanderbilt.edu (A.E.G.); hal.moses@vanderbilt.edu (H.L.M.); 4 Department of Biochemistry, Vanderbilt University, Nashville, TN 37232, USA; kimberly.n.johnson@vanderbilt.edu; 5 Research Medicine, Veterans Affairs, Tennessee Valley Healthcare System, Nashville, TN 37232, USA

**Keywords:** *TGFBR3*, TβRIII, Cancer-Associated-Fibroblasts (CAFs), tumor microenvironment, cancer stroma, Betaglycan

## Abstract

The TGF-β pathway plays a major role in tumor progression through regulation of epithelial and stromal cell signaling. Dysfunction of the pathway can lead to carcinoma progression and metastasis. To gain insight into the stromal role of the TGF-β pathway in breast cancer, we performed laser capture microdissection (LCM) from breast cancer patients and reduction mammoplasty patients. Microdissected tumor stroma and normal breast stroma were examined for gene expression. Expression of the TGF-β type III receptor (*TGFBR3*) was greatly decreased in the tumor stroma compared to control healthy breast tissue. These results demonstrated a 44-fold decrease in *TGFBR3* mRNA in tumor stroma in comparison to control tissue. We investigated publicly available databases, and have identified that *TGFBR3* mRNA levels are decreased in tumor stroma. We next investigated fibroblast cell lines derived from cancerous and normal breast tissue and found that in addition to mRNA levels, TβRIII protein levels were significantly reduced. Having previously identified that cancer-associated fibroblasts secrete greater levels of tumor promoting cytokines, we investigated the consequences of soluble-TβRIII (sTβRIII) on fibroblasts. Fibroblast conditioned medium was analyzed for 102 human secreted cytokines and distinct changes in response to sTβRIII were observed. Next, we used the fibroblast-conditioned medium to stimulate human monocyte cell line THP-1. These results indicate a distinct transcriptional response depending on sTβRIII treatment and whether it was derived from normal or cancerous breast tissue. We conclude that the effect of TβRIII has distinct roles not only in cancer-associated fibroblasts but that sTβRIII has distinct paracrine functions in the tumor microenvironment.

## 1. Introduction

There have been many advances in early detection, diagnosis and treatment of breast cancer, yet metastatic breast cancer remains a significant problem. Human breast cancer is a highly heterogeneous disease. Molecular profiling of whole tumor tissues has identified five major breast cancer phenotypes: Luminal A, Luminal B, Her2-like, Normal breast-like and Basal-like [1]. Furthermore, these breast cancer phenotypes correlate to patient survival with the luminal B subtype demonstrating the poorest overall outcome [2]. Recently, it has been shown that tumor progression depends not only on the intrinsic malignancy of the tumor cell, but also on the surrounding microenvironment, which is composed of immune cells, vasculature, fibroblasts and extracellular matrix. Using a cohort of 600 patients, de Kruijf and colleagues have evaluated the prognostic values of carcinoma stroma in breast cancer [3]. They demonstrated that tumor–stroma ratio in the primary tumor is a prognostic factor in early breast cancer patients. The stroma-rich tumor patients had a shorter overall survival in comparison to stroma-poor breast cancer patients [3]. In addition, stromal transcriptional differences within these subtypes have begun to be addressed. Progress on stromal molecular profiling is ongoing, but its depth is lagging behind the molecular profiling of the tumor epithelium. Almost a decade after tumor molecular profiling, Finak and colleagues have demonstrated that stromal signatures are important to pursue. Their molecular profiling of tumor stroma has revealed three subtypes of tumor stroma classified as: mixed outcome, good outcome and poor outcome [4]. This was based on a correlation between the overall patient outcome and stromal gene expression. A better understanding of the stromal gene expression changes could potentially offer new targets for therapy to prevent and suppress breast cancer progression, metastasis, and patient survival. Over the course of the past several years, it has become apparent that stromal cells adjacent to normal or transformed epithelium can significantly regulate the initiation and progression of cancer in vivo. Our attention has been focused on understanding the tumor microenvironment in the context of TGF-β signaling. Using mouse models to target the stroma, we have shown that loss of TGF-β signaling in FSP+ cells can lead to tumorigenesis of the adjacent epithelial cells [5] as well as promote carcinoma growth and invasion due to increased secretion of chemokines [6,7,8]. More recently, we have found unique roles in the stromal fibroblasts for tumor promotion by modification of the extracellular matrix [9]. We have also identified other components of the TGF-β family such as Bone Morphogenetic Proteins (BMP), and their tumor promoting role in carcinoma associated fibroblasts [10,11,12]. During these investigations, we discovered that a shared TGF-β component *TGFBR3* (also known as Betaglycan) was downregulated in Cancer-Associated-Fibroblasts (CAFs) [10]. *TGFBR3* has largely been studied in normal and cancerous cells and only recently investigated in tumor associated stromal cells [13]. *TGFBR3* is an interesting component of the TGF-β superfamily since it functions as a type III co-receptor not only for TGF-β signaling but also for BMP, Activins, Nodal, and GDF factors. The functional role of *TGFBR3* is controversial in breast cancer. Some reports suggest a tumor suppressive function of TβRIII [14], while other reports indicate a tumor-promoting role [15,16,17,18,19]. Currently there are no reports demonstrating the role of *TGFBR3* in the tumor microenvironment, despite the possibility that *TGFBR3* controversy in breast cancer might be influenced by tumor microenvironment.

The role of TGF-β in the stoma and fibroblasts has been extensively reviewed and investigated [20]. TGF-β can activate fibroblasts to become a fibrotic tumor promoting microenvironment, but can also result in tumor promotion when fibroblasts lack canonical components of the TGF-β signal transduction cascade [21]. What is clear from these studies is that intact, strict regulation of TGF-β signaling is required to maintain homeostasis not only in tumor cells but that of the surrounding tumor microenvironment.

Stromal–epithelial interactions offer new targets for adjuvant therapy to prevent and suppress human breast cancer progression. Current strategies to treat human breast cancer stroma are beginning to emerge, however, there are a significant number of unknown signaling pathways and targets left to be discovered [22]. We show that *TGFBR3* is absent in breast cancer stroma (fibroblasts), and that this correlates with increased cytokine expression. This expression can be resolved by restoring soluble *TGFBR3*. Clinical trials are currently underway with *TGFBR3* related peptides for the amelioration of fibrosis, and may soon find their way in to clinical trials for cancer patients [23].

## 2. Materials and Methods

### 2.1. Human Fibroblast Isolation and Culturing

Human fibroblast cell lines were established from fresh tumor and normal breast tissues obtained from de-identified patients (approved by Vanderbilt University Institutional Review Board, application IRB# 080603 “TGF-β in mammary development and tumorigenesis”). Tissue was washed in 15 mL of sterile DMEM F12 containing fungizone, gentamicin and penicillin streptomycin. Tissue was then transferred into a petri dish containing digestion media (DMEM 10% FCS, fungizone, gentamicin, penicillin streptomycin, collagenase, and hyaluronidase) where it was finely minced using sterile scalpel and scissors. Minced tissue was then transferred to sterile 50 mL conical tube containing additional 5 mL of fresh digestion media. Minced tissue and 15 mL of digestion media was place in 37 °C water bath shaker for 4 h. After 4 h of shaking/vortexing, digested tissue was centrifuged at 1000 rpm for 5 min. The remaining pellet was washed multiple times with DMEM F12, 10% fetal calf serum, fungizone, penicillin streptomycin, and gentamicin. The collected pellet was then plated in T25 flasks, in DMEM F12, supplemented with 10% fetal calf serum and antibiotics. Once colonies were formed partial trypsinization was performed to separate fibroblast from epithelial cells. Fibroblasts were then expended based on the experimental needs.

### 2.2. Protein Extraction, Western Blot and Cytokine Array

Total protein was isolated using Complete LysisM Buffer (Roche, Indianapolis, IN, USA). Protein was diluted to equal concentrations and equally loaded on 10% polyacrylamide gels prior to transfer to a nitrocellulose membrane. Protein concentration was determined using micro plate BCA assay (BioRad, Hercules, CA, USA). Blots were incubated overnight with Smad1 (Cell Signaling Cat#6944 1:1000), Smad 2 (Cell Signaling Cat#5339 1:1000), pSmad1/5 (Cell Signaling Cat#9516 1:1000), pSmad2 (Cell Signaling Cat#3108 1:1000), and Actin (Sigma Cat#A2066 1:4000) antibodies. HRP-conjugated secondary antibodies were used to visualize band intensity via x-ray film exposure using ECL western substrate (Perkin Elmer, Waltham, MA, USA). Conditioned medium was collected 48 h after equal cell numbers were plated into T-75 culture flasks. Supernatant was spun to remove cells and debris and 500 μL were used per membrane for Human cytokine array XL, performed following manufacturer instructions (RnD Systems Cat#ARY006) with assistance from VAPR (Vanderbilt Antibody Protein Resource). sTβRIII was no longer present in the conditioned medium collected, and was replaced with new medium after 48 h of treatment followed by an additional 48 h of medium conditioning, which was then collected. We did not alter the serum level or perform starvation.

### 2.3. Immunofluorescence

Immunofluorescence staining was performed with primary and secondary antibodies diluted in 12% Fraction-V BSA (Pierce, Thermofisher Scientific, Waltham, MA, USA) and slides were mounted in SlowFade mounting medium containing DAPI (Invitrogen, Carlsbad, CA, USA). All fluorescent secondary antibodies were highly cross-absorbed, produced in goat and used at a dilution of 1:200 for 20 min (Molecular Probes). The following primary antibodies were used: FAP (RnD Systems Cat#AF3715 1:200), NG2 (Millipore Cat#AB5320 1:200), PDGFRα (Cell Signaling Cat#5241 1:200), Vimentin (Covance Cat#PCK-594P 1:500), αSMA (Sigma Cat#A2547 1:500), FSP-1 (EMD Millipore Cat#07-2274), Phalloidin-594 (Molecular Probes Cat#A12381), and pSmad2 (Cell Signaling Cat#3108 1:500).

### 2.4. Laser Capture Microdissection and Expression Analysis

Laser Capture Microdissection (LCM) was performed on 5 μm sections derived from frozen breast core tumor biopsies (invasive ductal carcinomas) as well as tissues from normal breast reduction mammoplasties (both from de-identified patients). Furthermore, a trained pathologist has evaluated H&E sections of each sample prior to microdissection as to ensure proper collection of fibroblasts. The pathologist marked areas to collect. The criteria were as follows: fibroblast rich area of the stroma, without immune infiltrates present. LCM was performed on an Arcturus PixCell IIe microscope provided by Vanderbilt Translational Pathology Shared Resource. LCM-captured RNA was isolated using an RNAqueous-Micro kit (Ambion) and validated for array quality (Vanderbilt Genome Sciences Resource). Subsequent cDNA synthesis and amplification was completed by VANTAGE. Reactions were run in 96 well format with 10 ng of Total RNA used per reaction in the NUGEN FFPE Kit (Cat#3400-60, Lot#1009255-C). The reactions were run through First Strand and Second Strand synthesis, followed by 2 rounds of SPIA amplification to generate cDNA. The cDNA was frozen overnight at −20 °C and cleaned up the next day. The cDNA targets were quantitated on the Nanodrop. Overall the yields were robust, with an average yield of 10.2 µg providing enough amplified product.

### 2.5. RNA Preparation and Quantitative PCR (qPCR)

RNA was isolated and purified using an RNeasy Mini Kit and an RNase-Free DNase Set (Qiagen, Valencia, CA, USA). A total of 750 ng of RNA was used to synthesize cDNA using Superscript III reverse transcriptase as described by the manufacturer (Invitrogen, Carlsbad, CA, USA). BioRad iCycler and CFX96 machines were used for qPCR employing Power SYBR Green (Applied Biosystems) or SsoAdvanced SYBR Green Supermix (BioRad), respectively. Ct values were normalized to *GAPDH* for statistical analyses. Primer sequences are available in supplemental material (Table 1).

### 2.6. Statistical Analysis, Bioinformatics and Database Analysis

Analysis of gene expression correlating with RFS was performed using the kmplotter (http://kmplot.com). Kmplot.com uses more than 25 breast cancer gene expression databases to evaluate relapse free survival. Human gene symbols were entered into breast and JetSet probe selection was used to determine optimal representative microarray probe [24]. Automatic cutoff scores were selected during queries and RFS were selected. Statistical analysis was performed using Excel (Microsoft), Graphpad Prism (Graphpad Software, Inc., La Jolla, CA, USA), Statistical significance was deemed for any comparison where *p* ≤ 0.05.

## 3. Results

### 3.1. In Silico Analysis of TGF-β Pathway Related Genes Reveals TGFBR3 to Be Significantly Changed in Tumor Stroma

**Figure 1:**
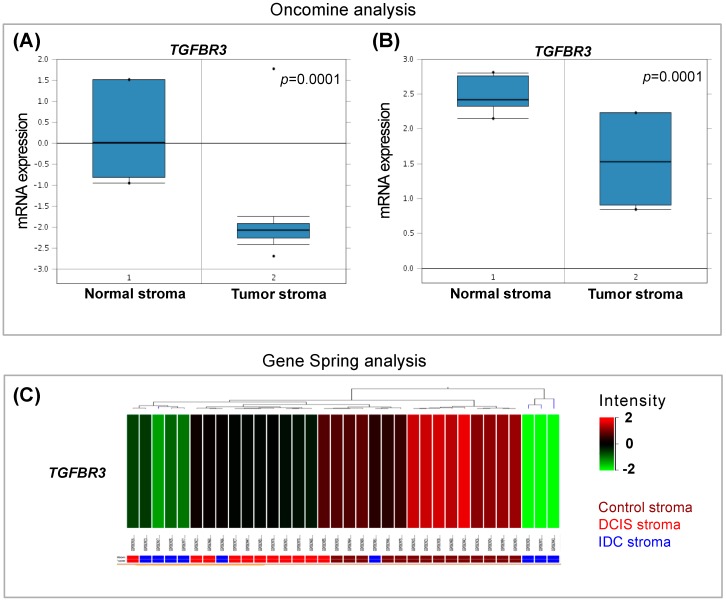
*TGFBR3* levels of expression in publicly available microarray datasets. (**A**, **B**) Oncomine transcriptome profiles yield results demonstrating a significant decrease in *TGFBR3* levels in the human breast tumor stroma; and (**C**) Ma et al. dataset consisting of stroma derived from normal, ductal carcinoma in situ (DCIS) and invasive ductal carcinoma (IDC) shows gradual decrease of *TGFBR3* from normal to IDC state. Note that these studies have not looked at *TGFBR3*.

Our laboratory has held a long-standing interest in TGF-β signaling, especially in the tumor microenvironment [25]. Utilizing publically available datasets, we analyzed the stroma of breast cancer patients for differences in gene expressions. Focusing on the genes in the TGF-β superfamily, we found that the most consistently changed gene expression in the tumor stroma among the TGF-β pathway related genes were a decrease in *TGFBR3* (Figure 1A–C). The *TGFBR3* levels were decreased in tumor stroma in 75% of patients while expression was intact across all the controls (Figure 1A).

Analyses of these stromal datasets suggest significant decrease in *TGFBR3* gene expression in human breast stroma. It is important to note that these studies have not specifically looked at *TGFBR3* expression changes. Figure 1A,B shows significant relative loss of *TGFBR3* in the invasive breast carcinoma stroma compared to the control stroma [4,26]. Interestingly, based on analysis of the Ma et al. dataset, it appears that this decrease in *TGFBR3* occurs gradually during breast cancer progression as demonstrated by the control stroma, ductal carcinoma in situ (DCIS) and invasive ductal carcinoma (IDC) (Figure 1C) [27].

**Figure 2:**
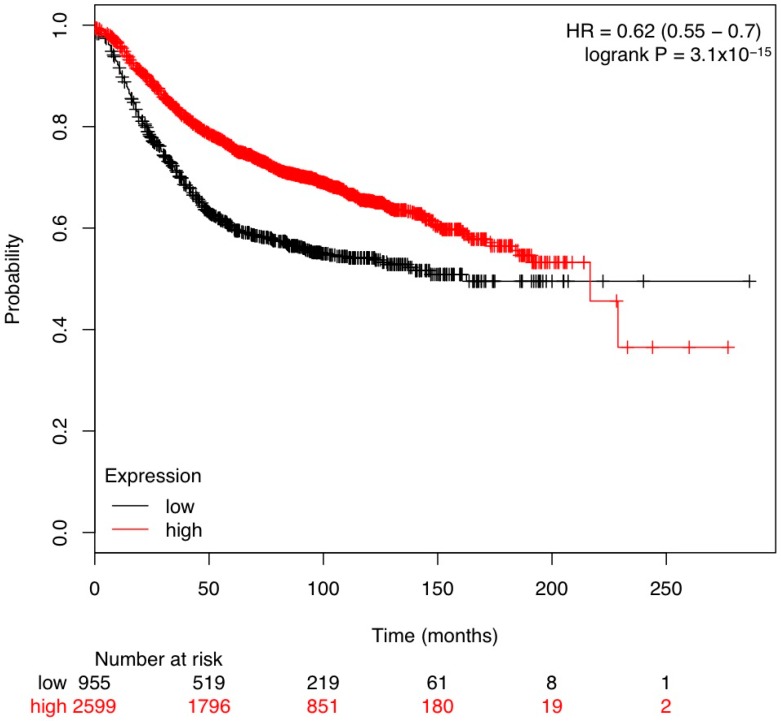
Kaplan–Meier analysis from kmplot.com of breast cancer patients with low *TGFBR3* expression levels (black) are linked to poorer survival compared to patients with high *TGFBR3* expression (red) (*p* < 0.01).

We next looked at databases that include survival from bulk tumors including the stroma and the tumor. Kaplan–Meier analysis for Relapse-Free-Survival (RFS) indicates a correlation of poor survival with lower *TGFBR3* gene expression (Figure 2).

**Figure 3:**
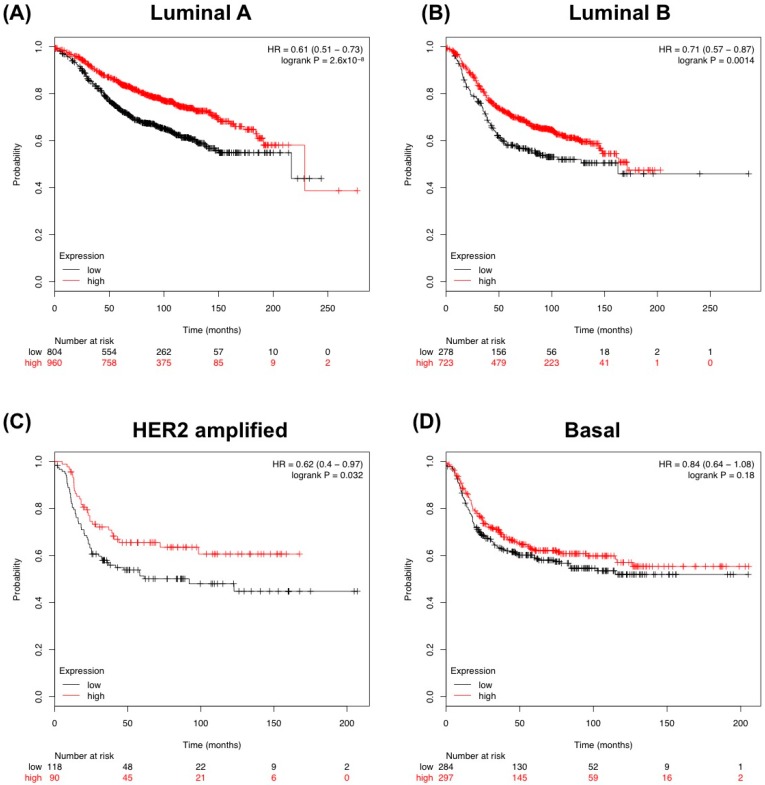
Relapse-Free Survival (RFS) analysis based on *TGFBR3* expression across molecular breast cancer subtypes. (**A**–**D**) Kmplot.com analysis using *TGFBR3* for each of the four distinct main molecular subtypes of breast cancer using Jetset probe, auto cut-off for threshold and censored at median.

We further looked at correlations of *TGFBR3* expression and RFS within molecular subtypes of human breast cancer. We found that three out of four subtypes investigated (Luminal A, Luminal B, HER2-amplified and Basal) had significantly higher correlation with poorer RFS (Figure 3A–C). Of interest was the effect of *TGFBR3* expression correlated with improved survival at 10 years (~120 months) in all breast cancer patients. However, for all patients (and specifically the luminal A and B subtypes) as time progressed, the expression correlation became less distinct towards 20 years. This possibly could be due to limited numbers. The decreased correlation with expression could potentially reflect the age of patients and other non-cancer related events. (Figure 2 and Figure 3).

### 3.2. Laser Capture Microdissection (LCM) of Human Tumor Stromal Cells

**Figure 4:**
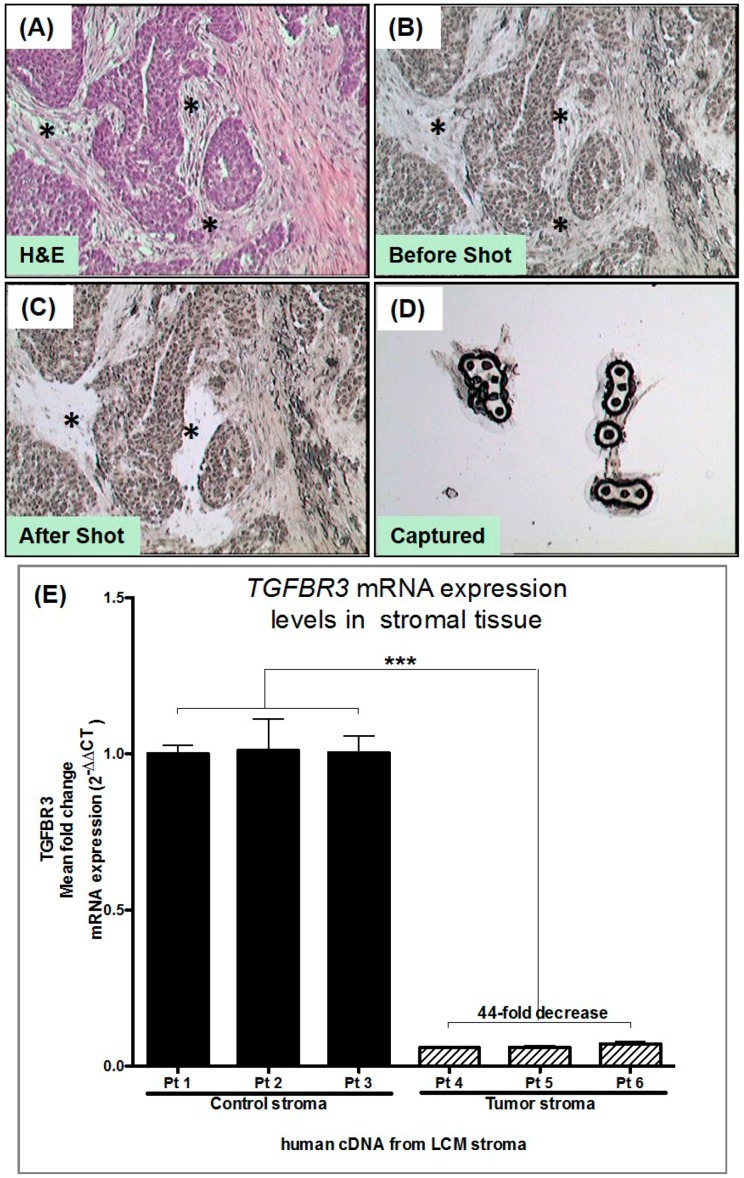
Laser capture microdissection (LCM) of human peritumoral stromal cells: (**A**) H&E stained frozen section of breast carcinoma; (**B**) unstained frozen section as seen in the LCM microscope before capture; (**C**) frozen section after capture showing absence of stromal cells; (**D**) captured stromal cells on LCM cap ready for RNA extraction; and (**E**) qPCR analysis of *TGFBR3* average mRNA expression (2^−ΔΔCt^) from stroma obtained from three patients with normal breast tissue and three patients with breast carcinoma. mRNA is normalized to *GAPDH* levels. Error bars indicate mean of four replicates with SEM error bars (*** *p* < 0.01 for unpaired two-tailed Student’s *t*-test).

We performed LCM to capture stroma from de-identified frozen tissues from breast cancer patients. For our control, we collected stroma from human breast reduction mammoplasty, which constituted our “normal stroma”. Previous studies have used different patients instead of so-called “adjacent normal”, as these patients’ cancer have significant inflammatory changes throughout the breast even with the absence of cancer in the field of view [4] (Figure 4A–D). We used LCM to enrich fibroblast collection by avoiding epithelial, immune cells and blood vessels. Pictures were taken before and after LCM to document the cells captured (Figure 4B,C) (RNA was isolated from the captured stromal cells, and cDNA was generated for qPCR). *TGFBR3* gene expression from three controls and three tumor-derived stroma demonstrated a 44-fold decrease in *TGFBR3* mRNA (Figure 4E). We validated that, as shown in Figure 1, *TGFBR3* is indeed transcriptionally downregulated in the stroma of breast cancer.

### 3.3. Human Breast Cancer-Associated Fibroblasts Have Reduced TGFBR3 mRNA and Protein

**Figure 5:**
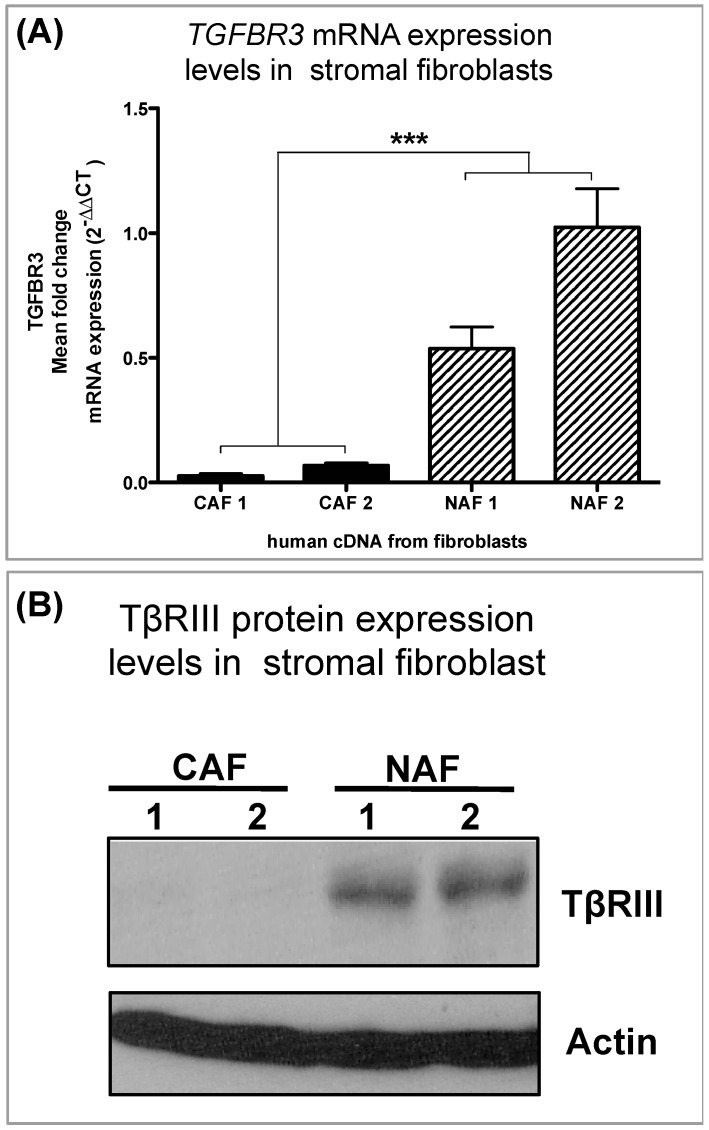
In vitro validation of *TGFBR3* protein and mRNA levels in human breast cancer fibroblast. (**A**) qPCR analysis of *TGFBR3* from human cDNA from cancer-associated fibroblasts (CAFs) and normal-associate fibroblasts (NAFs); graph bars represent the mean of three replicates with SEM error bars (*** *p* < 0.0001; for a one-way ANOVA). (**B**) Western blot analysis of TβRIII protein expression in CAFs and NAFs. mRNA is normalized to *GAPDH* levels and relative to control and fold changes are given in log^2^ scale. Mean of four replicates with SEM error bars (*** *p* < 0.01 for unpaired two-tailed Student’s *t*-test).

We generated a series of primary human fibroblast cell lines derived from either normal or cancerous breast tissue, which we have characterized previously [10]. These cell lines were not transformed and not used after a maximum of twenty passages. Cells were also monitored for contaminants and senescence and discarded if found. Interestingly, the in vitro cell line data for cancer associated fibroblasts (CAFs) and normal-associated fibroblasts (NAFs) demonstrate a decrease of *TGFBR3* in CAFs at both mRNA (Figure 5A) and protein levels (Figure 5B).

### 3.4. Human Breast Cancer Associated Fibroblasts and Normal Associated Fibroblasts Have Distinct Inflammatory Responses to Soluble TGFBR3

**Figure 6:**
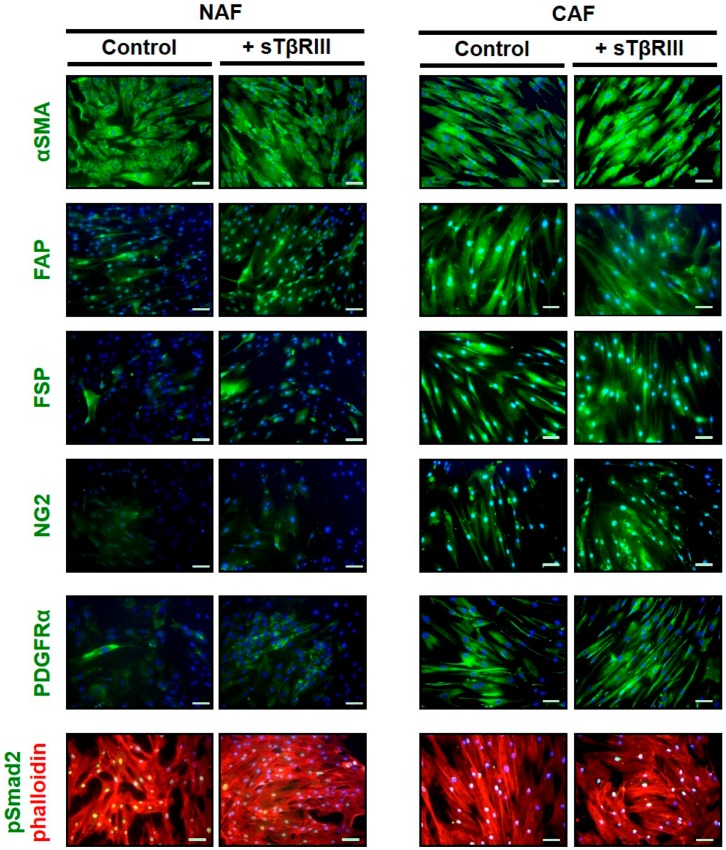
Immunofluorescence based characterization of Normal Associated Fibroblast (NAF) and Cancer Associate Fibroblast (CAF) cells upon sTβRIII treatment. NAF and CAF cells plated in chamber slides were stained for various markers of fibroblasts with and without treatment of 200 ng/mL of sTβRIII.

**Figure 7:**
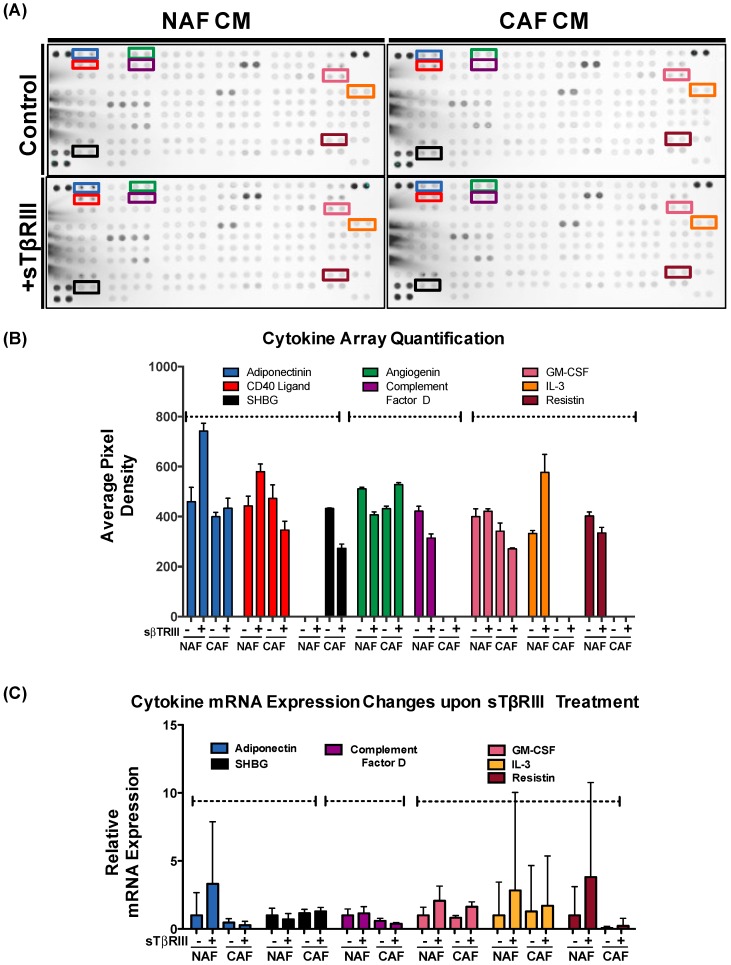
Human breast cancer-associated fibroblasts and normal-associated fibroblasts have distinct inflammatory responses to soluble *TGFBR3*. (**A**) Conditioned media was collected after 48 h after either no treatment or treatment with 200 ng/mL of soluble TβRIII (sTβRIII). Two hundred microliters of conditioned medium was used to analyze protein expression of 105 human cytokines on R&D Systems Human Cytokine Array XL detection kit. (**B**) Quantification of pixel intensity by IR secondary scanning revealed distinct pixel density in select cytokines with a specific response to sTβRIII treatment of either NAFs or CAFs. Column graph represents mean of pixel density value replicates with SD error bars. (**C**) qPCR for selected cytokines demonstrates relative mRNA expression (2^−ΔΔCt^) in cytokines from NAFs and CAFs treated with sTβRIII. mRNA is normalized to *GAPDH* levels and relative to control. The fold changes are given in log^2^ scale. Column graph represents mean of three replicates with SD error bars. No statistical significance was observed in qPCR analysis. Gene abbreviations are defined as follow: CD40 (cluster of differentiation), SHBG (sex hormone binding globulin), GM-CSF (granulocyte macrophage colony-stimulating factor), IL-3 (interleukin 3).

In addition to analyzing pivotal genes within TGF-β pathway, we also investigated secreted cytokines from the conditioned media of NAFs and CAFs. We chose to investigate the direct paracrine mechanism of *TGFBR3* (from CAFs), which entailed the shedding of the extracellular/soluble portion of the TβRIII protein. While gene expression data indicated a decrease of *TGFBR3* in the CAFs, we wanted to evaluate if the soluble protein (sTβRIII) could alter the signaling in the CAFs that were no longer expressing the gene. We have shown that TGF-β can regulate fibroblast-derived cytokines that can act directly upon the adjacent tumor/epithelium to contribute to carcinoma progression and metastasis [6,7,8]. Because loss of *TGFBR3* can be related to paracrine or cell autonomous signaling, we also investigated cytokine expression when NAFs and CAFs were treated with soluble form of *TGFBR3* (sTβRIII). We first tested for changes in fibroblast protein markers on NAFs and CAFs. We observed that while CAFs contained more positive staining for activation markers such as FAP and FSP-1, there was no appreciable change with sTβRIII treatment (Figure 6). We found that incubation with sTβRIII led to distinct changes in cytokines from either NAFs or CAFs in the conditioned media after 48 h (Figure 7A,B). Some cytokines only responded to sTβRIII in NAFs and not CAFs such as Adiponectin (Figure 7B). Other cytokines displayed opposite responses in NAFs compared to CAFs such as Angiogenein and CD40 ligand (Figure 7B). Other cytokines were only found in NAFs and were altered in the presence of sTβRIII such as Complement Factor D, Interleukin-3 (IL-3) and Resistin (Figure 7B). Only one cytokine was expressed in CAFs, which was Sex-hormone-binding globulin (SHBG) and expression was reduced with treatment of sTβRIII (Figure 7B). We next attempted to discern whether the treatment of sTβRIII on NAFs and CAFs was mirrored in mRNA transcription by qPCR, and found that the cytokines altered at the protein level (Figure 7B) were not significantly altered at the mRNA level (Figure 7C). Variability in many of the cytokines gene expression was directly related to a low presence of detectable transcript, even with relative high abundance of control housekeeping gene *GAPDH* (Figure 7C).

**Figure 8:**
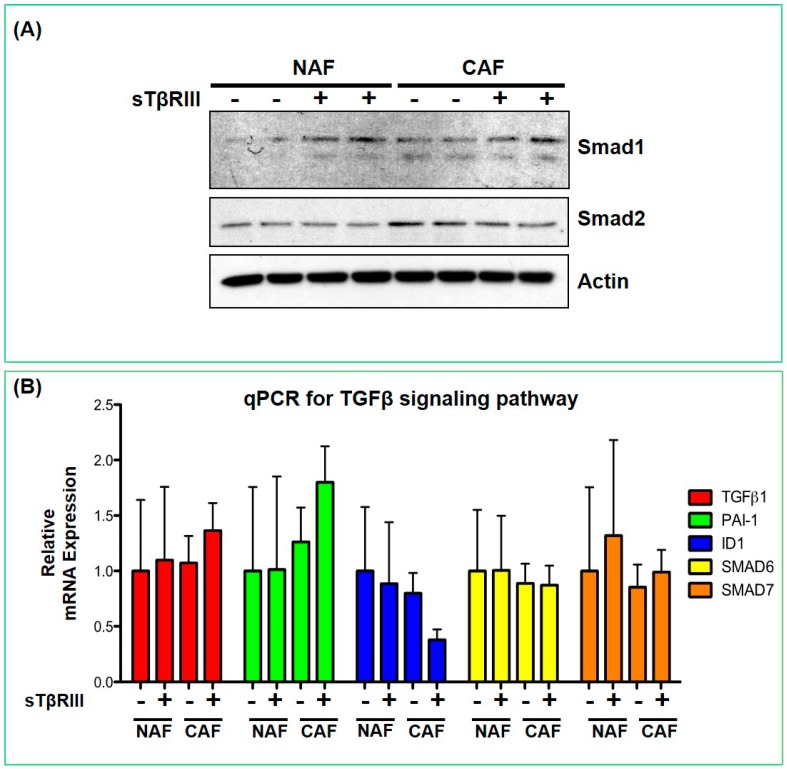
Validation of protein and mRNA changes in TGF-β signaling within NAFs and CAFs. (**A**) Western blot analysis of canonical Smad signaling in NAFs and CAFs treated with 200 ng/mL of sTβRIII for 24 h; and (**B**) qPCR for canonical TGF-β/BMP signaling components and targets.

We further investigated the effect of sTβRIII on canonical TGF-β signaling. Without any TGF-β ligand treatment, we saw no evidence of phosphorylation of Smads1, -2, -3, -5 and -9 (data not shown). We also did not see significant changes in the un-phosphorylated forms of Smad1 and Smad2 (Figure 8A). We also measured canonical transcriptional response genes of the TGF-β family via qPCR, and found that there were no significant changes with sTβRIII treatment after 24 h (Figure 8B). We conclude that the soluble form of *TGFBR3* has distinct function in its shed form than in its membrane-bound cell autonomous signaling.

### 3.5. Treatment of NAFs and CAFs with sTβRIII and Subsequent Conditioned Medium Alters Human Monocytes Cytokine Expression

**Figure 9:**
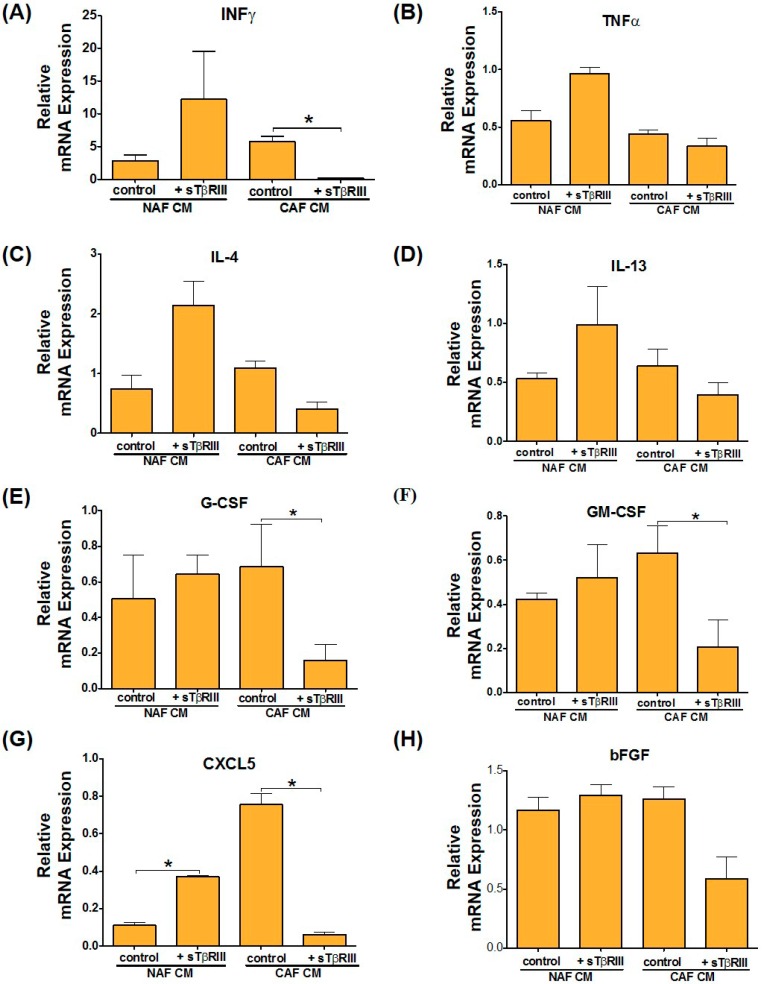
Treatment of THP-1 monocytes with NAF and CAF derived CM in the presence or absence of sTβRIII alters human monocytes cytokine expression. (**A**–**H**) qPCR from cDNA derived from THP-1 monocytes treated with 10% conditioned medium derived alone from NAFs and CAFs as well as upon 24 h treatment of NAFs and CAFs with sTβRIII. mRNA is normalized to *GAPDH* levels and relative to control. The fold changes are given in log 2 scale. Error bars indicate SEM. * *p* ≤ 0.05. Groups compared are indicated by black lines while statistically significant changes were marked by (*).

While fibroblasts are known to interact with many cell types normally and in cancer [28], here we show how the cytokines expressed from these NAFs and CAFs affected myeloid cells. CAFs have been routinely shown to regulate tumor suppression or promotion via paracrine activity with the immune system [29]. To evaluate gene expression associated with immune activation and inflammation we treated the human monocyte cell line THP-1 with NAF and CAF derived conditioned media (CM) in the presence or absence of sTβRIII. The results indicate that upon treatment of THP-1 cells with NAF CM +/− sTβRIII or CAF CM—sTβRIII there is a pattern towards an increase of that *INFγ*. However, when treated with CAF CM+ sTβRIII, there is a significant decrease in *INFγ* gene expression (Figure 9A).

Expression of *TNFα* had a significant decrease after both NAF and CAF CM treatment, however only NAF CM + sTβRIII restored *TNFα* expression to the levels observed in the untreated control (Figure 9B). Furthermore, we looked at gene expression of *IL-4* and *IL-13*, which typically promote alternative activation of macrophages into M2 cells, which is generally tumor promoting. Interestingly, we found that in comparison to treatment with NAF CM, addition of sTβRIII to NAF CM shows significant increase in *IL-4* and *IL-13* levels, while CAF CM + sTβRIII reduced *IL-4* and *IL-13* gene expression, although not statistically significant (Figure 9C,D). Gene expression of the activation markers *G-CSF* and *GM-CSF* showed a trending decrease across all treatments compared to controls; however, only cells treated with CAF CM in the presence of sTβRIII showed significant reduction (Figure 9E,F). Finally, since we had not activated these cells into macrophages or polarized them directly, we looked at the activation makers, *CXCL5* and *bFGF* to determine if the cells were differentiating upon treatment with CM. *CXCL5* results indicate significant and opposite expression changes when THP-1 cells were treated with fibroblast-derived CM from NAF and CAF in the presence of sTβRIII. Treatment with NAFs CM+ sTβRIII has increased expression levels, while treatment with CAFs CM+ sTβRIII demonstrates decreased *CXCL5* expression levels in comparison to treatment with CM without sTβRIII (Figure 9G). Furthermore, activation of *CXCL5* was more suppressed by NAF CM than CAF CM relative to the untreated control (Figure 9G). We did not observe any significant changes in *bFGF* expression, and only a trending decrease in CAF CM in the presence of sTβRIII. Overall, these results suggest a potential paracrine cross-talk between fibroblasts, secreted factors and monocytes. Treating CAFs that lack *TGFBR3* with sTβRIII appears to have decreased inflammatory ability, which could potentially lead to suppression of myeloid tumor promoting cell activity.

## 4. Discussion

Xenograft and genetic studies in mice have been able to provide useful information regarding stromal epithelial interactions that regulate adjacent carcinoma initiation and progression. In the mammary gland, it has now been shown that human mammary fibroblasts have the capacity to suppress tumorigenesis of adjacent epithelium [26]. In our studies related to TGF-β signaling, it has become clear that stromal fibroblasts have the ability to suppress or initiate carcinomas in adjacent normal epithelium [5,30]. Furthermore, we have found that within an initiated tumor microenvironment, TGF-β signaling in stromal fibroblasts can have a profound influence upon tumor progression. Together the results, obtained through modified stromal TGF-β signaling in mice with those obtained using human fibroblast and epithelial cell recombination, suggest that stromal–epithelial interactions can have a significant impact on the regulation of adjacent carcinoma initiation and progression in vivo. It has been demonstrated that loss of TGFBR2, specifically within the stroma, results in enhanced tumor progression. This highlights the previous work demonstrating the tumor suppressive functions of the canonical TGF-β pathway [31]. While fibroblasts can be relatively simple to grow, those derived from human breast cancer patients have been notoriously difficult for our laboratories and others to establish and maintain. These studies are limited due to technical challenges revolving around establishing fibroblast cell lines. Once access to fibroblast cell lines are established within breast cancer scientific community, studies focused on expression of unique domains of *TGFBR3* will be possible to pursue. This will aid an in-depth dissection of the function of both, the membrane bound and soluble *TGFBR3*, in the CAFs.

Since our focus was to gain a better insight into TGF-β pathway, we chose to do laser capture microdissection (LCM) of tumor stroma. Investigating TGF-β signaling components led to the finding that *TGFBR3* was markedly decreased in tumor stroma compared with control stroma. *TGFBR3* loss was the most consistent change among all the TGF-β pathway genes. To verify these results, we have performed quantitative real-time PCR analysis on LCM samples for *TGFBR3*. After linking this data to clinical outcome in publicly available microarray data sets, we have determined that there is correlation between *TGFBR3* expression levels and patient outcome. Specifically, the loss of *TGFBR3* gene expression is linked to poor outcome.

To date, the functional context of *TGFBR3* remains controversial in breast cancer, where studies report both tumor suppressive and tumor-promoting functions [14,32,33]. In cancer cells it has been reported that *TGFBR3* interacts with the p38 signaling pathway to coordinate disseminated tumor cells with TGF-β maintained in a stem/quiescent state. Blocking this process with *TGFBR3* could be one way to restore proliferation and growth at secondary metastatic sites after dormancy [34]. Currently, there are limited reports demonstrating the role of *TGFBR3* in the tumor microenvironment. The most significant discoveries of stromal loss of *TGFBR3* have been in dendritic cells, whereby the microenvironment becomes more immune-tolerant of the tumor [13]. One possibility for the conflicting role of *TGFBR3* in breast cancer might be the influence of the tumor microenvironment. Since inactivating mutations in the gene encoding *TGFBR3* have not been reported, we suspect reduced expression through epigenetic or other unknown signaling mechanisms are responsible. Further experiments that investigate how tumors and their genetic alterations influence *TGFBR3* will be useful in many types of cancers. Our study provides new insight to the role of *TGFBR3* in the tumor microenvironment and perhaps helps resolve the current controversy of *TGFBR3*’s role in cancer. Addressing the function of *TGFBR3* in the tumor microenvironment could help us determine if the current data on *TGFBR3* in tumor is context dependent and that the behavior of the tumor cell is modified/regulated by the extracellular *TGFBR3* or shed/soluble *TGFBR3*. Previous work understanding the mechanisms and triggers for the shedding and creation of sTβRIII demonstrates the complexity of these paracrine mechanisms [35]. Reports of sTβRIII anti-tumorigenic effects on cancer cells via inhibition of cell motility and invasion have been very informative about the capacity of sTβRIII to restore “normal” TGF-β signaling [36,37,38,39]. We show here that this “restoration” is not in play in CAFs, and their ability to integrate sTβRIII signaling is distinct from tumor cells. This may not pose a pro or anti fibrotic treatment as idealized in fibroblasts with TGF-β therapeutics.

Despite the fact that *TGFBR3* is important for delivering TGF-β ligands to receptors and initiation of TGF-β signaling, this molecule is still an understudied component of the tumor microenvironment. *TGFBR3* has the potential to be a new key player in the tumor microenvironment, thus it is important to further characterize the physiological function of *TGFBR3* in tumor–stroma interaction. The relationship of *TGFBR3* to other key tumor–stroma molecular mediators is an essential direction for providing an insight into tumor microenvironment and determining novel options for early diagnosis and anticancer therapy [40]. Recently, a role for *TGFBR3* was found in the tumor microenvironment specifically in dendritic cells. Loss of *TGFBR3* resulted in a tumor immune tolerant microenvironment. Addition of sTβRIII was found to reactivate the immune response and suppress T-regulatory cells that are key mediators of immune tolerance [13]. We find that distinct cytokines from CAFs and NAFs are altered as well when treated with sTβRIII, with a loss of endogenous *TGFBR3* (Figure 7). We explored the effect of the fibroblast paracrine secretions to monocytes, but the diversity and immense number of immune and other cells that could be affected by the loss or addition of *TGFBR3* should be expanded with further research.

A clinical trial investigating blocking TGF-β interaction with *TGFBR3* to prevent fibrosis was completed in 2007 (NCT ID: NCT00656825) [41]. This trial was encouraged by the finding that a peptide named P144 could block the interaction of *TGFBR3* with ligand activation necessary for fibrosis [42,43]. Furthermore this peptide has been shown to be effective at blocking TGF-β directed Epithelial-to-Mesenchymal Transition (EMT), the cancer stem cell phenotype and progression of metastasis [44]. Additionally, these peptides have been shown to limit angiogenesis and neovascularization, which could lead to anti-angiogenic therapies [45,46]. Whether blocking *TGFBR3* and TGF-β signaling with peptides or the use of shed/soluble *TGFBR3* will likely be determined on the context of the TGF-β activity in the disease. We find that when *TGFBR3* is lost, it is detrimental, and restoration of this signaling component will be key to improving targeted therapies. Further distinctions between *TGFBR3* and ligands/receptors as well as intracellular cell autonomous signaling still await important discoveries.

In addition to our focus on *TGFBR3*, gene-expression profiling of adjacent tumor stroma can provide information to enhance the prediction of clinical outcome in comparison to the current approaches performed in pathology. The stroma gene expression signatures could then be translated and used for development of tests that could serve as a guide for more informed clinical decision-making. We are in need of new strategies to use as either a preventive measure or for treatment of the metastatic disease. Stromal–epithelial interactions offer new targets for adjuvant therapy to prevent and suppress human breast cancer progression. Current strategies to treat human breast cancer are predominantly focused on targeting the carcinoma cell population specifically; however, there are a significant number of patients that will develop distant metastases even though standard therapies are applied. At present, the five-year survival rate for breast cancer is low when distant metastases are detected at the time of primary tumor diagnosis, and it is likely that conventional therapy in addition to targeting adjacent supporting cell populations would improve long-term survival within this patient population. An enhanced understanding of the stromal contribution to breast cancer initiation and progression will be necessary to target both the cancer cells and the microenvironment for effective treatment of metastatic disease.

## Figures and Tables

**Table 1:** Human SYBR Primer Sequences. Forward and reverse primer sequences used are listed and either sourced from validated sequences from Harvard primer bank or NCBI Primer tool. All sequences are optimized for 60 °C melting temperature and result in a single product by melting curve analysis.

GENE	FWD SEQUENCE	REV SEQUENCE
***TGFBR3***	TGGGGTCTCCAGACTGTTTTT	CTGCTCCATACTCTTTTCCGGG
***ADIPONECTIN***	AACATGCCCATTCGCTTTACC	TAGGCAAAGTAGTACAGCCCA
***COMPLEMENT FACTOR D***	GACACCATCGACCACGACC	GCCACGTCGCAGAGAGTTC
***GM-CSF***	TCCTGAACCTGAGTAGAGACAC	TGCTGCTTGTAGTGGCTGG
***IL-3***	TCAACAGGGCTGTCAAGAGTT	CAGATAGAACGTCAGTTTCCTCC
***RESISTIN***	CTGTTGGTGTCTAGCAAGACC	CCAATGCTGCTTATTGCCCTAAA
***SHBG***	GCCCAGGACAAGAGCCTATC	CCTTAGGGTTGGTATCCCCATAA
***INFγ***	TCGGTAACTGACTTGAATGTCCA	TCGCTTCCCTGTTTTAGCTGC
***TNFα***	GAGGCCAAGCCCTGGTATG	CGGGCCGATTGATCTCAGC
***IL-4***	CGGCAACTTTGTCCACGGA	TCTGTTACGGTCAACTCGGTG
***IL-13***	GAAGGCTCCGCTCTGCAAT	TCCAGGGCTGCACAGTACA
***G-CSF***	GCTGCTTGAGCCAACTCCATA	GAACGCGGTACGACACCTC
***CXCL5***	AGCTGCGTTGCGTTTGTTTAC	TGGCGAACACTTGCAGATTAC
***bFGF***	AGAAGAGCGACCCTCACATCA	CGGTTAGCACACACTCCTTTG
***TGFβ1***	CAATTCCTGGCGATACCTCAG	GCACAACTCCGGTGACATCAA
***PAI-1***	GACATCCTGGAACTGCCCTA	GGTCATGTTGCCTTTCCAAGT
***ID1***	CTGCTCTACGACATGAACGG	GAAGGTCCCTGATGTAGTCGAT
***SMAD6***	CCTCCCTACTCTCGGCTGTC	GGTAGCCTCCGTTTCAGTGTA
***SMAD7***	CCAACTGCAGACTGTCCAGA	CAGGCTCCAGAAGAAGTTGG
***GAPDH***	CTGGGCTACACTGAGCACC	AAGTGGTCGTTGAGGGCAATG
